# Connected-SegNets: A Deep Learning Model for Breast Tumor Segmentation from X-ray Images

**DOI:** 10.3390/cancers14164030

**Published:** 2022-08-20

**Authors:** Mohammad Alkhaleefah, Tan-Hsu Tan, Chuan-Hsun Chang, Tzu-Chuan Wang, Shang-Chih Ma, Lena Chang, Yang-Lang Chang

**Affiliations:** 1 Department of Electrical Engineering, National Taipei University of Technology, Taipei 10608, Taiwan; 2 Division of General Surgery, Cheng Hsin General Hospital, Taipei 112, Taiwan; 3 Department of Communications, Navigation and Control Engineering, National Taiwan Ocean University, Keelung 202301, Taiwan

**Keywords:** breast tumor segmentation, convolutional neural network, deep learning, X-ray images

## Abstract

**Simple Summary:**

The segmentation of breast tumors is an important step in identifying and classifying benign and malignant tumors in X-ray images. Mammography screening has proven to be an effective tool for breast cancer diagnosis. However, the inspection of breast mammograms for early-stage cancer can be a challenging task due to the complicated structure of dense breasts. Several deep learning models have been proposed to overcome this particular issue; however, the false positive and false negative rates are still high. Hence, this study introduced a deep learning model, called Connected-SegNets, that combines two SegNet architectures with skip connections to provide a robust model to reduce false positive and false negative rates for breast tumor segmentation from mammograms.

**Abstract:**

Inspired by Connected-UNets, this study proposes a deep learning model, called Connected-SegNets, for breast tumor segmentation from X-ray images. In the proposed model, two SegNet architectures are connected with skip connections between their layers. Moreover, the cross-entropy loss function of the original SegNet has been replaced by the intersection over union (IoU) loss function in order to make the proposed model more robust against noise during the training process. As part of data preprocessing, a histogram equalization technique, called contrast limit adapt histogram equalization (CLAHE), is applied to all datasets to enhance the compressed regions and smooth the distribution of the pixels. Additionally, two image augmentation methods, namely rotation and flipping, are used to increase the amount of training data and to prevent overfitting. The proposed model has been evaluated on two publicly available datasets, specifically INbreast and the curated breast imaging subset of digital database for screening mammography (CBIS-DDSM). The proposed model has also been evaluated using a private dataset obtained from Cheng Hsin General Hospital in Taiwan. The experimental results show that the proposed Connected-SegNets model outperforms the state-of-the-art methods in terms of Dice score and IoU score. The proposed Connected-SegNets produces a maximum Dice score of 96.34% on the INbreast dataset, 92.86% on the CBIS-DDSM dataset, and 92.25% on the private dataset. Furthermore, the experimental results show that the proposed model achieves the highest IoU score of 91.21%, 87.34%, and 83.71% on INbreast, CBIS-DDSM, and the private dataset, respectively.

## 1. Introduction

The United States of America reported a total of 43,250 female deaths and 530 male deaths due to breast cancer in 2022 [1]. Researchers are motivated by these statistics to develop accurate tools for early breast cancer diagnosis, which will offer physicians more options for treatment. Mammograms are still being widely used to detect the presence of any abnormalities in breasts [2,3,4]. Mammogram images show different types of breast tissues as pixel clusters with different intensities [5]. These tissues include fiber-glandular, fatty, and pectoral muscle tissues [6]. On mammography, abnormal tissues such as lesions, tumors, lumps, masses, or calcifications may be indicators of breast cancer [7,8]. However, there is always the possibility of human error when analyzing and diagnosing breast cancer due to dense breasts and the high variability between patients [9,10,11]. Additionally, mammography screening sensitivity is affected by image quality and radiologist experience [12,13].

Automated techniques are being developed to analyze and diagnose breast mammograms with the goal of counteracting this variability and standardizing diagnostic procedures [14,15]. The rapid emergence of artificial intelligence (AI) and deep learning (DL) has significant implications for breast cancer diagnosis [16,17,18]. The advancements in image segmentation using convolutional neural networks (CNNs) have been applied to segment breast cancer from X-ray images [19,20,21,22,23]. The earlier works on mass segmentation faced some challenges, such as low signal to noise ratio, indiscernible mass boundaries, high false positives, and high false negative rates. To address these challenges, one study proposed a deeply supervised UNet model (DS U-Net) coupled with dense conditional random fields (CRFs) for lesion segmentation from whole mammograms [19]. The DS U-Net model has produced a Dice score of 79% on the INbreast dataset and 83% on the CBIS-DDSM dataset, whereas its IoU score is 83% and 86% on the INbreast and CBIS-DDSM datasets, respectively. Another study [20] proposed an attention-guided dense up-sampling network (AU-Net) for accurate breast mass segmentation from mammograms. An asymmetrical encoder–decoder structure is employed in this AU-Net and it uses an effective up-sampling block and attention-guided dense up-sampling block (AU block). The AU block is designed to have three merits. First, dense upsampling compensates for the information loss experienced during bilinear up-sampling. Second, it integrates high- and low-level features more effectively. Third, it highlights channels with rich information via the channel attention function. Compared to the state-of-the-art FCNs, AU-Net achieved the best performance, with a Dice score of 90% on the INbreast dataset and 89% on the CBIS-DDSM dataset.

However, such models do not capture the features of different scales of masses effectively, and therefore they suffer from low segmentation accuracy. Hence, a new model, called UNet, was presented to mitigate the limitations of the previous models [21]. UNet integrates the high-level features of the encoder with the low-level features of the decoder. Through skip connections, the UNet architecture was able to maintain this form of fusion for a variety of medical applications. The UNet architecture achieves better performance on different biomedical segmentation applications. Asma Baccouche et al. [22] introduced Connected-UNets to segment breast masses. This method integrated atrous spatial pyramid pooling (ASPP) in the two standard UNets. The architecture of Connected-UNets was built on the attention network (AUNet) and residual network (ResUNet). To augment and enhance the images, cycle-consistent generative adversarial networks (CycleGANs) were used between two unpaired datasets. Additionally, a regional deep learning approach called you-only-look-once (YOLO) has been used to detect breast lesions from mammograms. Finally, a full-resolution convolutional network (FrCN) has been implemented to segment breast lesions. The Connected-UNets model has produced a Dice score of 94% and 92% on the INbreast and CBIS-DDSM datasets, respectively. Moreover, it has achieved an IoU score of 90% and 86% on INbreast and CBIS-DDSM, respectively. Badrinarayanan et al. [23] proposed a practical deep fully convolutional neural network architecture for semantic pixel-wise segmentation, termed SegNet. Its segmentation architecture consists of an encoder network and a decoder network followed by a pixel-wise classification layer. Topologically, the architecture of the encoder network matches that of the 13 convolutional layers in the VGG16 network. The role of the decoder network is to map the low-resolution encoder feature maps to full-input-resolution feature maps for pixel-wise classification. The SegNet model has achieved satisfactory segmentation performance. However, since the SegNet architecture does not consist of skip connections, incorporating fine multiscale information during the training process is challenging.

**Figure 1:**
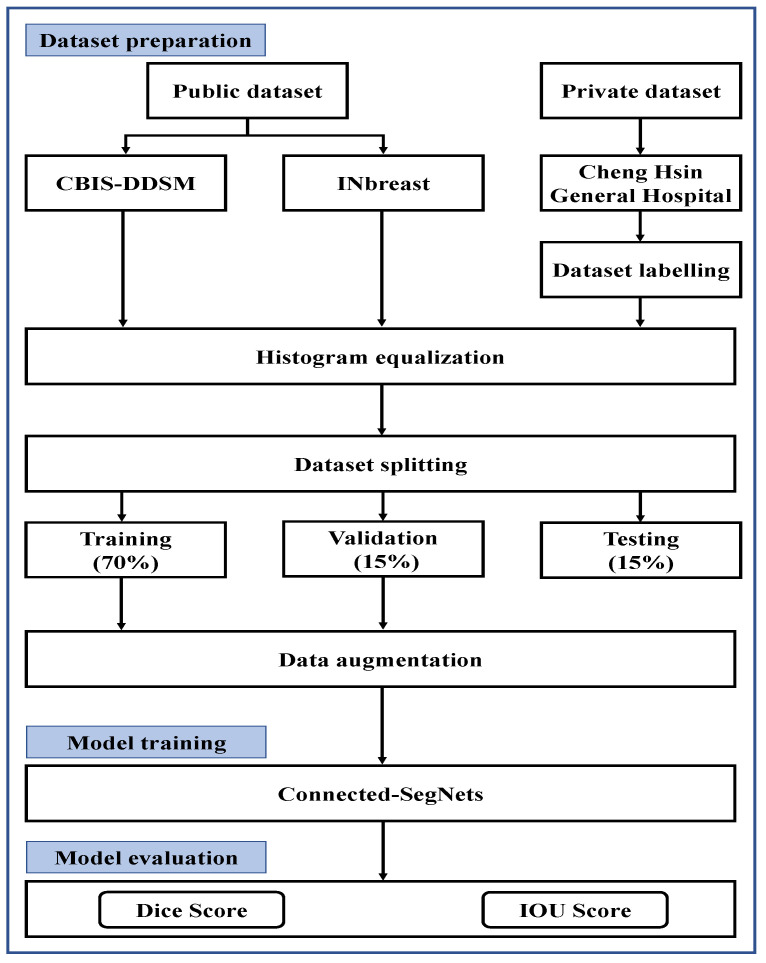
Flow chart of the proposed tumor segmentation system.

This study combines the characteristics of the Connected-UNets and SegNet models to form Connected-SegNets from two standard SegNets with skip connections for breast tumor segmentation from breast mammograms. The flow chart of the proposed system is illustrated in Figure 1. The major contributions of this study include the following.

1. This study proposes a deep learning model called Connected-SegNets for breast tumor segmentation from X-ray images.
2. The proposed model, Connected-SegNets, is designed using skip connections, which helps to recover the spatial information lost during the pooling operations.
3. The original SegNet cross-entropy loss function has been replaced by the IoU loss function to overcome any noisy features and enhance the detection of the false negative and false positive cases.
4. The histogram equalization method of the contrast limit adapt histogram equalization (CLAHE) is applied to all datasets to enhance the compressed areas and smooth the pixel distribution.
5. Image augmentation methods including rotation and flipping have been used to increase the number of training data and to reduce the impact of overfitting.

The rest of this paper is organized as follows. Section 2 describes the datasets and architectural details of the proposed method. Section 3 presents the experimental results. Section 4 discusses the merits of this study. Finally, the article is concluded with its primary findings in Section 5.

## 2. Materials and Methods

This research uses the two publicly available datasets of INbreast and CBIS-DDSM, and one private dataset obtained from Cheng Hsin General Hospital in Taiwan. Initially, a histogram equalization, CLAHE, is applied to all datasets to enhance the compressed areas and smooth the pixel distribution. Then, each X-ray dataset is randomly divided into 70%, 15%, and 15% for training, validation, and testing, respectively. Finally, the training and validation samples are augmented to increase the amount of data before feeding them to the proposed Connected-SegNets model.

### 2.1. Datasets

The proposed model, Connected-SegNets, has been evaluated on the following datasets.

#### 2.1.1. INbreast Dataset

The INbreast dataset is a collection of mammograms from Centro de Mama Hospital de S. João, Breast Centres Network, Porto, Portugal. A total of 410 images with 115 cases were collected from August 2008 to July 2010 [24,25], and 95 of 115 cancer cases involved both breasts in women. Four different types of breast diseases are recorded in the database, including calcification, mass, distortions, and asymmetries. This database includes images from craniocaudal (CC) and mediolateral oblique (MLO) perspectives. Moreover, the breast density is divided into four categories according to the breast imaging reporting and data system (BI-RADS) assessment categories, which are: entirely fat (BI-RADS 1), scattered fibroglandular (BI-RADS 2), heterogeneously dense (BI-RADS 3), and extremely dense (BI-RADS 4). All the images were saved in two sizes: 3328×4084 or 2560×3328 pixels. Among the 410 mammograms, 107 images contain breast tumors. Hence, these 107 images were selected for this study. The 107 images were randomly split into 90 images for training and 17 images for testing, as shown in Table 1. The image augmentation methods, including rotation and flipping, were applied to the training data. The augmentation methods increased the number of breast tumor mammography images to 720 images. The 720 images were randomly split into 576 images for training data and 174 images for validation data, as shown in Table 2.

#### 2.1.2. CBIS-DDSM Dataset

The DDSM is a public dataset provided by the University of South Florida Computer Science and Engineering Department, Sandia National Laboratories, and Massachusetts General Hospital [26]. The CBIS-DDSM is an updated and standardized version of the DDSM [27]. It contains a variety of pathologically verified cases, including malignant, benign, and normal cases. DDSM is an extremely useful database for the development and testing of computer-aided diagnosis (CAD) systems due to its scale and the ground truth validation it offers. The CBIS-DDSM collection includes a subset of the DDSM data organized by expert radiologists. It also comprises pathological diagnosis, bounding boxes, and region of interest (ROI) segmentation for training data. Among all mammography images with tumors in the CBIS-DDSM dataset, 838 images were selected for this study. The 838 images were randomly split into 728 images for training data and 110 images for testing data, as shown in Table 1. The image augmentation methods, including rotation and flipping, were applied to the training samples. Through image augmentation, the number of breast tumor mammography images was increased to 5824. The 5824 images were randomly split into 4659 images for training data and 1165 images for validation data, as shown in Table 2.

#### 2.1.3. Private Dataset

The private dataset comprised mammography images from the Cheng Hsin General Hospital, Taipei City, Taiwan. Initially, VGG image annotator (VIA) software was used by an expert radiologist from the department of medical imaging to mark the tumor location based on the pathological data [28]. Then, all the labeled images were verified and confirmed by the department of hematology and oncology. Finally, the dataset was de-identified for patient privacy. A total of 196 mammography images were collected from January 2019 to December 2019. All the mammograms consist of tumors with a grade of breast imaging reporting and data system assessment category 4 (BIRADS 4) or higher. A total of 196 mammography images were randomly split into 148 images for training and 48 images for testing, as shown in Table 1. The image augmentation methods, including rotation and flipping, were applied to the training samples. Through image augmentation methods, the number of breast tumor mammography images was increased to 1184. The 1184 images were randomly split into 947 images for training and 237 images for validation, as shown in Table 2.

### 2.2. Data Preprocessing

This research study only focused on the segmentation step. Initially, the ROI of the tumor was cropped manually. The ROI of the tumor was resized into 256×256. In order to eliminate additional noise and degradation caused by the scanning process of digital X-ray mammography, all images were preprocessed [29,30].

#### 2.2.1. Histogram Equalization

**Figure 2:**
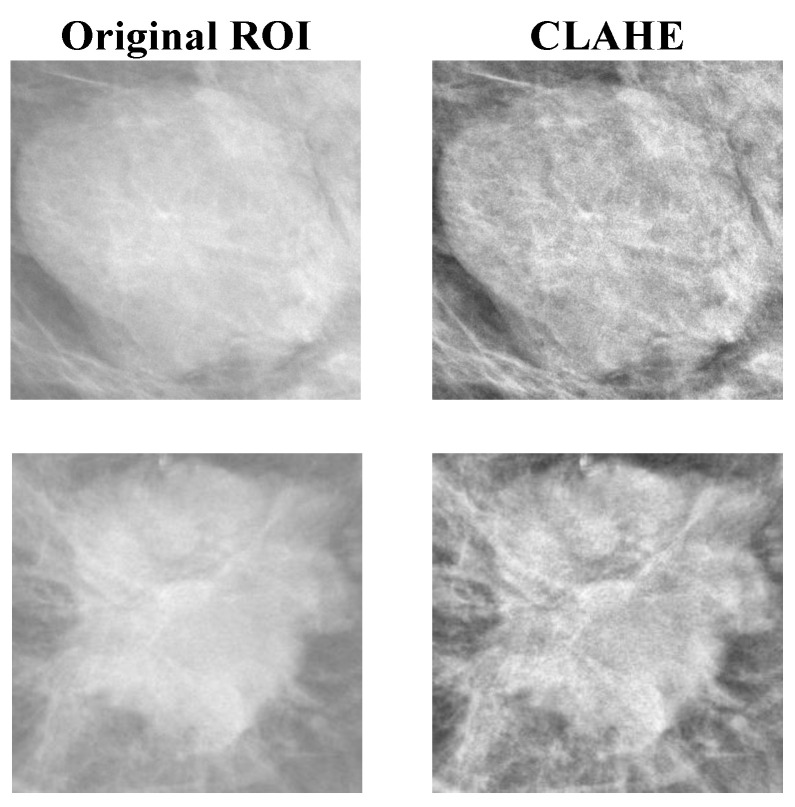
Sample results after applying the histogram equalization (CLAHE) to random ROI images from the datasets.

Histogram equalization is a well-known technique widely used for contrast enhancement [31]. It is used in a variety of applications, including medical image processing and radar signal processing, due to its simple function and effectiveness [32,33,34,35]. Histogram equalization well distributes the pixels over the full dynamic intensity range. One drawback of histogram equalization is that the background noise can be increased when the image is too bright or too dark in the local area after the histogram equalization, which is mainly due to the flattening property of the histogram equalization. This study applied the local histogram equalization method called CLAHE to address the above challenges. CLAHE is an adaptive extension of histogram equalization. It helps in the dynamic preservation of the local contrast features of an image. CLAHE has been applied to all datasets of this study. The sample results on the datasets after applying the CLAHE are shown in Figure 2. From Figure 2, it is noted that the edges of the tumors became clearer after applying the CLAHE technique. A total of 107, 838, and 196 ROIs were obtained from the INbreast, CBIS-DDSM, and the private datasets, respectively. The complete details of the mammography datasets are listed in Table 1.

#### 2.2.2. Image Augmentation

**Figure 3:**
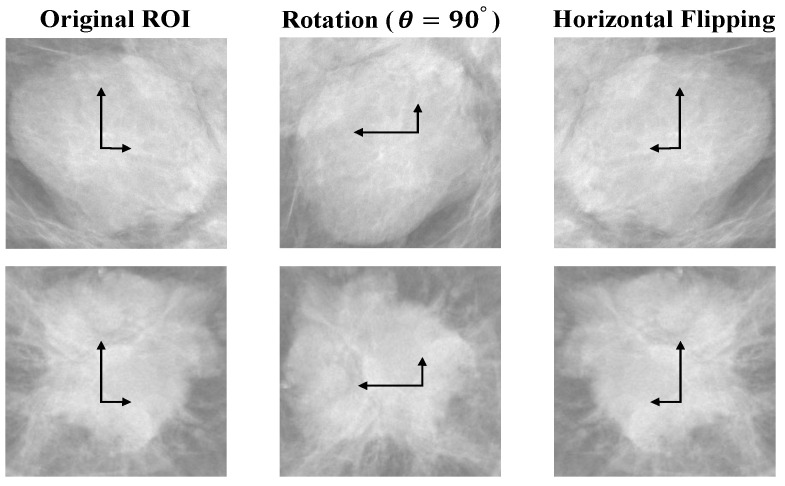
Random sample results after applying the rotation and flipping augmentation methods on the original ROIs. Arrows refer to the direction of the image.

The most common problem that DL models might face is the overfitting problem due to the limited amount of training samples [36,37,38]. As a result of overfitting, a model might detect or classify features derived from the training samples, but the same model will not be able to detect or classify features derived from unseen samples. To address the issue of overfitting, this study has used two image augmentation methods, namely rotation and flipping. First, bi-linear interpolation has been used to rotate each image around its center point by a value of 90° degrees counter-clockwise up to 360°. By using the bi-linear interpolation method, the rotated image has the same aspect ratio as the original image, without losing any part of the image. Second, mirroring or flipping is the simplest augmentation approach. It results in a dataset with twice as many images. The flipping technique is basically the same as the rotation technique; however, it transforms rotation in the reverse direction. The sample results on the datasets after applying the augmentation methods are shown in Figure 3.

The raw ROIs of the training data were augmented by rotating at an angle of 90° and horizontal flipping. Hence, a total of 720, 5824, and 1184 ROIs were generated from the INbreast, CBIS-DDSM, and private datasets, respectively. Then, the data were randomly split into training and validation. Detailed information of the mammography datasets in terms of the training data is provided in Table 2.

### 2.3. Proposed Model

**Figure 4:**
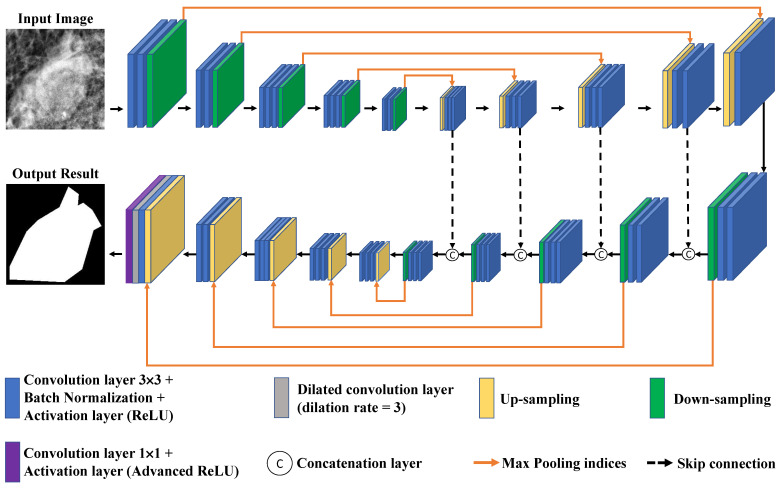
Architecture of the proposed Connected-SegNets model.

SegNet can record pooling indices when applying Max pooling. These pooling indices are used to up-sample the images to the original size. Hence, the required graphics processing unit (GPU) memory for training the model can be lower. Inspired by the success of SegNet and Connected-UNets, this research proposed a model, called Connected-SegNets, which connects two standard SegNets using additional adapted skip connections. The overall architecture of the proposed Connected-SegNets model is shown in Figure 4.

The proposed model consists of two encoder and two decoder networks. The first decoder network and the second encoder network are connected with additional skip connections after cascading a second SegNet. This helps to recover the fine-grained features that are lost in the encoding of the SegNet and apply them to encode the high-resolution features by connecting them to the previously decoded features. The proposed Connected-SegNets architecture is deepened by stacking two SegNets. The upper half of the proposed architecture is similar to SegNet, which uses the first 13 convolutional layers in the VGG16 network as the encoder network [39]. In the decoder network, the last convolutional layer is removed. Each encoder network comprises two convolutional kernels, which includes 3×3 convolutional layers followed by an activation rectified linear unit (ReLU) and a batch normalization (BN) layer. Then, a maximum pooling indices operation is applied to the output of each encoder network before passing the information to the next encoder. Each decoder network consists of a 2×2 transposed convolution unit that is concatenated with the previous encoder output, and then the result is fed into two convolution blocks, which consist of 3×3 convolutions followed by an activation ReLU and a BN layer. Additionally, a second SegNet is attached to the first SegNet through new skip connections that use information from the first up-sampling pathway. The result of the last decoder block is concatenated with the same result after being fed into a 3×3 convolution layer followed by an activation ReLU and a BN layer. This serves as the input of the first encoder network to the second SegNet. The output of the maximum pooling indices operations of each of the three encoder networks is fed into 3×3 convolution layers and then concatenated with the output of the last previous decoder network. The result is next down-sampled to the next encoder network. Finally, the last output is given to a dilation layer with a dilation rate of 3, followed by an advanced ReLU activation layer to generate the predicted mask. In order to obtain more features, a dilation layer with a dilation rate of 3 is used in the last layer. Moreover, an activation ReLU limits the maximum value to 1, which is called an advanced ReLU. The details of the Connected-SegNets layers are listed in Table 3.

### 2.4. Experimental Environment and Parameter Settings

All experiments were performed using a PC with an Intel i7-9700K CPU, 55 GB of DDR4 RAM, and an NVIDIA GeForce RTX 2080Ti GPU with 11 GB of memory. The software environment used a Windows 10 64-bit operating system, python 3.8.12, CUDA 10.1, cuDNN 7.6.5, and TensorFlow 2.8.0. The learning rate was set to 0.0001 using the Adam optimizer [40] and the batch size was 4. The loss function was the IoU loss function.

### 2.5. Evaluation Metrics

In this research, precision, recall, IoU score, and Dice score evaluation metrics have been used to evaluate the proposed model based on the confusion matrix. The confusion matrix is an evaluation metric often used to evaluate classification, detection, and segmentation algorithms. The confusion matrix shows information about the true classes and the predicted classes. The true class and the predicted class can be positive or negative. The true negative (TN) case is when both the true case and the predicted case are tumors. False negatives (FN) occur when the true case is not a tumor, but the predicted case is. The false positive (FP) case occurs when the true case is a tumor while the prediction is a non-tumor. True positives (TP) occur when the actual case is non-tumor and the predicted case is tumor. The Dice score is also known as the F1-score, which represents the harmonic mean of precision and recall, as expressed in Equation (3). Additionally, the IoU evaluation metric represents the percentage of overlap between the predicted classes and the true classes, as represented in Equation (4).

(1) $$Precision=\frac{TP}{TP+FP}$$

(2) $$Recall=\frac{TP}{TP+FN}$$

(3) $$Dice\;score=2\times \frac{Precision\times Recall}{Precision+Recall}$$

(4) $$IoU\;score=\frac{TP}{TP+FP+FN}$$

## 3. Results

### 3.1. Results on INbreast Dataset

The confusion matrix results of Connected-SegNets on the INbreast dataset are listed in Table 4. From the Table 4, it is observed that the proportion of actual tumors that was correctly identified as tumors (TP) by Connected-SegNets is 96%. This is the highest TP rate compared to the other datasets. In addition, the proportion of non-tumors that was correctly identified as non-tumors (TN) by Connected-SegNets is 88%.

### 3.2. Results on CBIS-DDSM Dataset

The identification results of Connected-SegNets on the CBIS-DDSM dataset are listed in Table 5. From the Table 5, it can be seen that the proportion of true tumors that was correctly identified as tumors (TP) by Connected-SegNets is 93%. Moreover, the proportion of non-tumors that was correctly identified as non-tumors (TN) by Connected-SegNets is 87%.

### 3.3. Results on Private Dataset

The results of the Connected-SegNets model on the private dataset are listed in Table 6. It is observed that the proportion of actual tumors that was correctly identified as tumors (TP) by Connected-SegNets is 92%. On the other hand, the proportion of tumors that were not tumors and were correctly identified as non-tumors (TN) by Connected-SegNets is 89%. This TN rate is considered to be the highest compared to other datasets.

**Figure 5:**
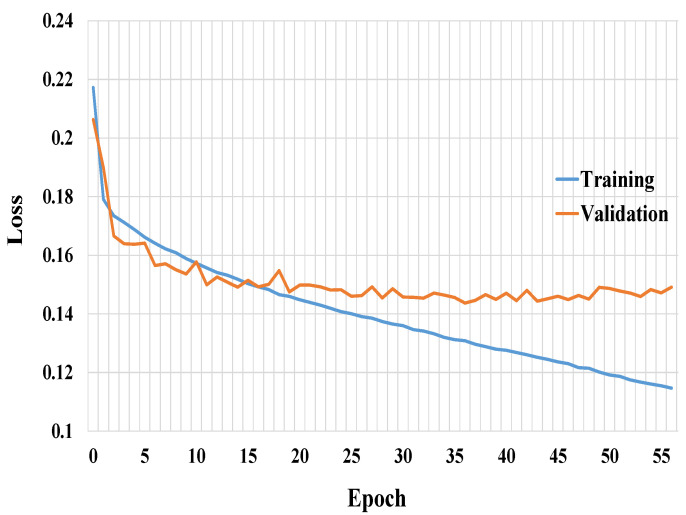
The training and validation accuracy curves of Connected-SegNets.

**Figure 6:**
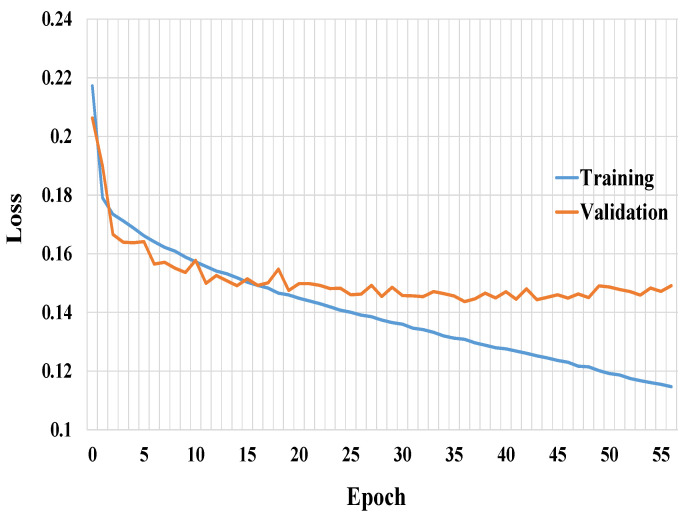
The training and validation loss curves of Connected-SegNets.

The accuracy and loss curves of the training and validation for Connected-SegNets are shown in Figure 5 and Figure 6, respectively. It can be noted from Figure 5 and Figure 6 that the training and validation curves behave similarly, which is an indication that the proposed Connected-SegNets can be generalized and does not suffer from overfitting.

A large number of epochs might cause a deep learning model to overfit the data, whereas a small number of epochs can lead to smooth convergence. Therefore, the early stop technique has been utilized during the model training to avoid overfitting. The validation dataset is used to track the model training performance. The early stop method can help to set a suitable training epoch by tracking the best performance on the validation dataset. Therefore, when the validation performance stops improving, an early stop mode of the training process will be activated. Moreover, using the early stop algorithm not only can avoid the overfitting problem, but it also can help with choosing the optimal hyperparameter configurations for training the model. The early stop algorithm steps are shown in Algorithm 1. In this research, the validation tracking, ActStepSetting, was set to 20 iterations. Hence, if the validation performance did not improve after 20 iterations, the training was stopped automatically.
**Algorithm 1** Validation Loss Tracking for Early Stop**Input:** LatestValLoss, ActStepSetting**Output**: BestValLossScore1: EarlyStop
←False;
2: **if**
BestValidationRepeatNum<=ActStepSetting
**then**
3:
    **if** LatestValLoss<BestValLossScore **then**
4:      BestValidationRepeatNum←0;5:      BestValLossScore←LatestValLoss;6:    **else**7:      BestValidationRepeatNum←BestValidationRepeatNum+1;8:    **end if**
9: **else**
10:    EarlyStop←True;
11: **end if**
12: **return** (BestValLossScore)


### 3.4. Comparison of Segmentation Results

As shown in Table 7, the segmentation results of each testing datum were evaluated by the two evaluation metrics, Dice score and IoU score, for the segmented maps per pixel, and compared with the original ground truth. It is noted that the proposed Connected-SegNets model produced the highest Dice score of 96.34%, 92.86%, and 92.25% on the INbreast, CBIS-DDSM, and private datasets, respectively. Moreover, the proposed model achieved the highest IoU Score of 91.21%, 87.34%, and 83.71% on the INbreast, CBIS-DDSM, and private datasets, respectively. Finally, the comparative results show that the proposed model, Connected-SegNets, outperformed the related models in terms of Dice score and IoU score on the three datasets.

**Figure 7:**
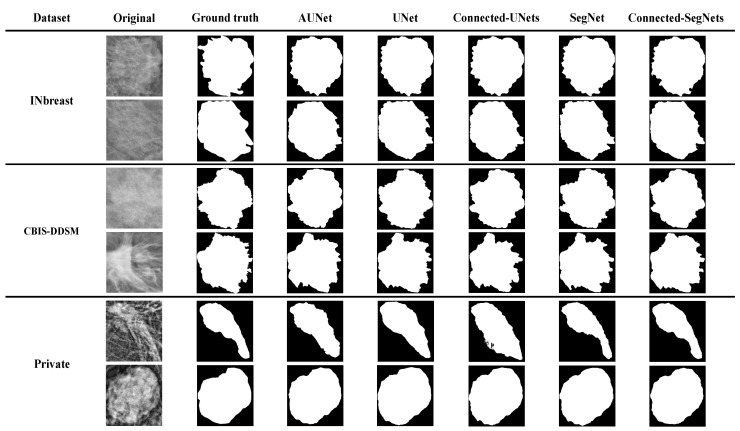
Example of the breast tumor segmentation results using AUNet, UNet, Connected-UNets, SegNet, and the proposed Connected-SegNets on the testing data of INbreast, CBIS-DDSM, and the private dataset.

Figure 7 shows some examples of the segmented ROI results generated by different models against their ground truth images. It is clearly observed that the quality of the segmentation maps of the Connected-SegNets model contain less error and produce more precise segmentation compared to other methods.

## 4. Discussion

In recent years, several DL models have been developed and applied for breast tumor segmentation. These DL models have achieved remarkable success in segmenting breast tumors in mammograms. Nevertheless, many of these DL models produce high false positive and false negative rates [41]. The SegNet model is considered to be one of the deep learning models that is easy to modify and further optimize to provide better segmentation performance in different fields. Therefore, this study proposed a DL model, called Connected-SegNets, based on SegNet, for better breast tumor segmentation. The main goal of the proposed Connected-SegNets model is to improve the overall performance of breast tumor segmentation. Hence, several techniques have been implemented and incorporated into the proposed method in order to achieve this goal. These techniques include deepening the architecture with two SegNets, replacing the cross-entropy loss function of the standard SegNet with the IoU loss function, applying histogram equalization (CLAHE), and performing image augmentation. Figure 7 illustrates the segmentation results of AUNet, Standard UNet, Connected-UNets, Standard SegNet, and the proposed Connected-SegNets on the testing data of the INbreast, CBIS-DDSM, and private datasets. The segmentation results of the proposed Connected-SegNets are the closest to the ground truth compared to those of the AUNet, UNet, Connected-UNets, and SegNet models. The proposed model fully connects two single SegNets using additional skip connections. These are helpful to recover the spatial information that is lost during the pooling operations. Moreover, the IoU loss function leads to a more robust model. Furthermore, the histogram equalization (CLAHE) has been applied to smoothen the distribution of the image pixels for better pixel segmentation. Additionally, image augmentation methods, including rotation and flipping, have been applied to increase the number of training samples and reduce the impact of overfitting. This has led to more accurate segmentation performance compared to the other models. The significant improvement is shown in Table 4, Table 5 and Table 6, where the Connected-SegNets model has the TP value of 96%, 93%, and 92%, on the INbreast, CBIS-DDSM, and private datasets, respectively. Similarly, the TN value is of 88%, 87%, and 89%, on INbreast, CBIS-DDSM, and the private dataset, respectively. The results of the proposed model, Connected-SegNets, showed a significant segmentation improvement compared to the other models, with a maximum Dice score of 96.34% on the INbreast dataset, 92.86% on the CBIS-DDSM dataset, and 92.25% on the private dataset. Similarly, the Connected-SegNets model has achieved the highest IoU score of 91.21% on the INbreast dataset, 87.34% on the CBIS-DDSM dataset, and 83.71% on the private dataset. Overall, the proposed Connected-SegNets model has outperformed DS U-Net, AUNet, UNet, Connected-UNets, and SegNet in terms of Dice score and IoU score. This shows the power of the proposed model to learn complex features through the connections added between the two SegNets in the proposed Connected-SegNets, which take advantage of the decoded features as another input in the encoder pathway.

## 5. Conclusions

This research proposed a deep learning model, namely Connected-SegNets, for breast tumor segmentation from X-ray images. Two SegNets were used in the proposed model, both of which were fully connected via additional skip connections. The cross-entropy loss function of the original SegNet was replaced by the IoU loss function to make the proposed model more robust against sparse data. Additionally, the contrast limit adapt histogram equalization (CLAHE) was applied to enhance the compressed areas and smooth the pixel distribution. Moreover, two augmentation methods including rotation and flipping were used to increase the number of training samples and prevent overfitting. The experimental results showed that Connected-SegNets outperformed the existing models, with the highest Dice scores of 96.34%, 92.86%, and 92.25%, and the highest IoU scores of 91.21%, 87.34%, and 83.71% on the INbreast, CBIS-DDSM, and private datasets, respectively. Future work will focus on implementing new deep learning algorithms for tumor detection and classification for automatic breast cancer diagnosis.

## Figures and Tables

**Table 1:** Distribution of the mammography datasets.

Dataset	Raw ROIs	Training Samples	Testing Samples
INbreast dataset	107	90	17
CBIS-DDSM dataset	838	728	110
Private dataset	196	148	48
Total	1141	966	175

**Table 2:** The number of training and validation samples before and after data augmentation.

Dataset	Raw Images	Augmented Images	Training	Validation
INbreast Dataset	90	720	576	144
CBIS-DDSM dataset	728	5824	4659	1165
Private dataset	148	1184	947	237
Total	966	7728	6182	1546

**Table 3:** The detailed architecture of the proposed Connected-SegNet.

	SegNet1				
No.	Layer Name	Output	Filter Size	No. of Filters	No. of Layers
1	Input	256 × 256 × 1			1
2	Conv1	256 × 256 × 64	3 × 3	64	2
3	Maxpool ^1^	128 × 128 × 64			1
4	Conv2	128 × 128 × 128	3 × 3	128	2
5	Maxpool ^1^	64 × 64 × 128			1
6	Conv3	64 × 64 × 256	3 × 3	256	3
7	Maxpool ^1^	32 × 32 × 256			1
8	Conv4	32 × 32 × 512	3 × 3	512	3
9	Maxpool ^1^	16 × 16 × 512			1
10	Conv5	16 × 16 × 512	3 × 3	512	3
11	Maxpool ^1^	8× 8 × 512			1
12	Upsampling ^2^	16 × 16 × 512			1
13	Conv6	16 × 16 × 512	3 × 3	512	3
14	Upsampling ^2^	32 × 32 × 512			1
15	Conv7	32 × 32 × 512	3 × 3	512	2
16	Conv8	32 × 32 × 256	3 × 3	256	1
17	Upsampling ^2^	64 × 64 × 256			1
18	Conv9	64 × 64 × 256	3 × 3	256	2
19	Conv10	64 × 64 × 128	3 × 3	128	1
20	Upsampling ^2^	128 × 128 × 128			1
21	Conv11	128 × 128 × 128	3 × 3	128	2
22	Conv12	128 × 128 × 64	3 × 3	64	1
23	Upsampling ^2^	256 × 256 × 64			1
24	Conv13	256 × 256 × 64	3 × 3	64	1
25	Conv13	256×256×64			
26	Conv14	256×256×64	3×3	64	2
27	Maxpool ^1^	128×128×64			1
28	Concatenate	128×128×128			1
29	Conv15	128×128×128	3×3	128	2
30	Maxpool ^1^	64×64×128			1
31	Concatenate	64×64×256			1
32	Conv16	64×64×256	3×3	256	3
33	Maxpool ^1^	32×32×256			1
34	Concatenate	16×16×512			1
35	Conv17	32×32×512	3 × 3	512	3
36	Maxpool ^1^	16×16×512			1
37	Concatenate	16×16×1024			1
38	Conv18	16×16×512	3 × 3	512	3
39	Maxpool ^1^	8×8×512			1
40	Upsampling ^2^	16×16×512			1
41	Conv19	16×16×512	3×3	512	3
42	Upsampling ^2^	32×32×512			1
43	Conv20	32×32×512	3×3	512	2
44	Conv21	32×32×256	3×3	256	1
45	Upsampling ^2^	64×64×256			1
46	Conv22	64×64×256	3×3	256	2
47	Conv23	64×64×128	3×3	128	1
48	Upsampling ^2^	128×128×128			1
49	Conv24	128×128×128	3×3	128	2
50	Conv25	128×128×64	3×3	64	1
51	Upsampling ^2^	256×256×64			1
52	Conv26	256×256×64	3×3	64	1
53	Conv27	256×256×64	3 × 3 (D ^3^ = 3)	64	1
54	Output	256×256×1	1×1	1	1

^1^ Maxpooling: Maxpooling and recording of the indices. ^2^ Upsampling: Upsampling with the recorded indices. ^3^ D: Dilation rate.

**Table 4:** Confusion matrix results of the proposed Connected-SegNets on INbreast dataset.

Connected-SegNets			
		**Ground Truth**	
		Tumor	Non-Tumor
**Prediction**	Tumor	96% (TP)	4% (FN)
	Non-Tumor	12% (FP)	88% (TN)

**Table 5:** Confusion matrix results of the proposed Connected-SegNets on CBIS-DDSM dataset.

Connected-SegNets			
		**Ground Truth**	
		Tumor	Non-Tumor
**Prediction**	Tumor	93% (TP)	7% (FN)
	Non-Tumor	13% (FP)	87% (TN)

**Table 6:** Confusion matrix results of the proposed Connected-SegNets on the private dataset.

Connected-SegNets			
		**Ground Truth**	
		Tumor	Non-Tumor
**Prediction**	Tumor	92% (TP)	8% (FN)
	Non-Tumor	11% (FP)	89% (TN)

**Table 7:** Comparison results between the proposed Connected-SegNets and the related segmentation models on the testing datasets of INbreast, CBIS-DDSM, and the private dataset, respectively.

Model	INbreast Dataset		CBIS-DDSM Dataset		Private Dataset	
	Dice Score (%)	IoU Score (%)	Dice Score (%)	IoU Score (%)	Dice Score (%)	IoU Score (%)
DS U-Net [19]	79.00	83.40	82.70	85.70	NA	NA
AUNet [20]	90.12	86.51	89.03	82.65	89.44	80.87
UNet [21]	92.14	88.23	90.47	84.79	89.11	80.21
Connected-UNets [22]	94.45	89.72	90.66	85.81	90.41	81.33
SegNet [23]	92.01	88.77	90.52	85.30	88.49	81.97
**Connected-SegNets**	**96.34**	**91.21**	**92.86**	**87.34**	**92.25**	**83.71**

## Data Availability

The data presented in this study are available in this article.
