# Correction: Alkhaleefah et al. Connected-SegNets: A Deep Learning Model for Breast Tumor Segmentation from X-ray Images. *Cancers* 2022, *14*, 4030

**DOI:** 10.3390/cancers15082237

**Published:** 2023-04-11

**Authors:** Mohammad Alkhaleefah, Tan-Hsu Tan, Chuan-Hsun Chang, Tzu-Chuan Wang, Shang-Chih Ma, Lena Chang, Yang-Lang Chang

**Affiliations:** 1 Department of Electrical Engineering, National Taipei University of Technology, Taipei 10608, Taiwan; muhai@ntut.edu.tw (M.A.);; 2 Division of General Surgery, Cheng Hsin General Hospital, Taipei 112, Taiwan; 3 Department of Communications, Navigation and Control Engineering, National Taiwan Ocean University, Keelung 202301, Taiwan

In the original publication [1], there was a mistake in Figure 5 as published. Figure 6 was repeated twice. The corrected Figure 5 appears below.

The authors state that the scientific conclusions are unaffected. This correction was approved by the Academic Editor. The original publication has also been updated with the correct Figure 5.

## Figures and Tables

**Figure 5:**
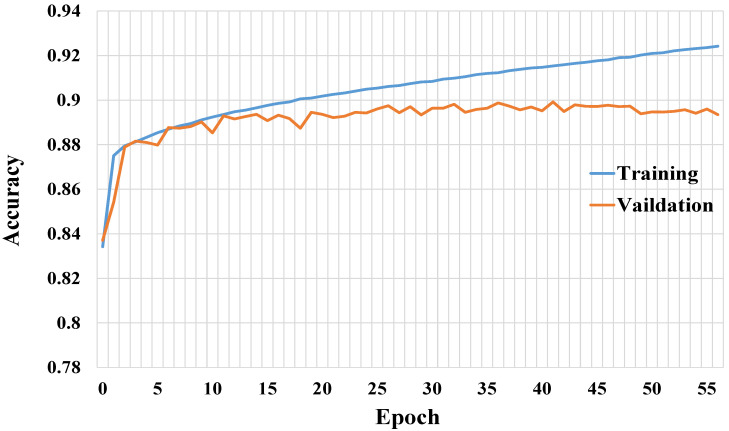
The training and validation accuracy curves of Connected-SegNets.
